# Preliminary assessment of cardiac short term safety and efficacy of manganese chloride for cardiovascular magnetic resonance in humans

**DOI:** 10.1186/1532-429X-13-6

**Published:** 2011-01-14

**Authors:** Juliano L Fernandes, Pippa Storey, Jose Alvaro da Silva, Gabriel S de Figueiredo, Jose M Kalaf, Otavio R Coelho

**Affiliations:** 1 Department of Internal Medicine - University of Campinas - Unicamp - R. Antonio Lapa 1032 - Campinas - Brazil; 2 Center for Biomedical Imaging - New York University - 660 First Avenue 4th floor - New York - USA; 3 Department of Radiology - Radiologia Clinica de Campinas - Av Jose de Souza Campos 870 - Campinas - Brazil

## Abstract

**Background:**

Manganese based agents are intracellular and accumulate inside myocytes allowing for different imaging strategies compared to gadolinium contrasts. While previous agents release manganese very slowly in the circulation, MnCl_2 _allows for rapid Mn2^+ ^uptake in myocytes, creating a memory effect that can be potentially explored. Data on animal models are very encouraging but the safety and efficacy of this approach in humans has not yet been investigated. Therefore, our objectives were to study the safety and efficacy of a rapid infusion of manganese chloride (MnCl_2_) for cardiovascular magnetic resonance (CMR) in humans.

**Methods:**

Fifteen healthy volunteers underwent a CMR scan on a 1.5 T scanner. Before the infusion, cardiac function was calculated and images of a short axis mid-ventricular slice were obtained using a 2D and 3D gradient-echo inversion recovery (GRE-IR) sequence, a phase-sensitive IR sequence and a single breath-hold segmented IR prepared steady-state precession acquisition for T_1 _calculations. MnCl_2 _was infused over three minutes at a total dose of 5 μMol/kg. Immediately after the infusion, and at 15 and 30 minutes later, new images were obtained and cardiac function re-evaluated.

**Results:**

There was a significant decrease in T_1 _values compared to baseline, sustained up to 30 minutes after the MnCl_2 _infusion (pre,839 ± 281 ms; 0 min, 684 ± 99; 15 min, 714 ± 168; 30 min, 706 ± 172, P = 0.003). The 2D and 3D GRE-IR sequence showed the greatest increase in signal-to-noise ratio compared to the other sequences (baseline 6.6 ± 4.2 and 9.7 ± 5.3; 0 min, 11.3 ± 4.1 and 15.0 ± 8.7; 15 min, 10.8 ± 4.0 and 16.9 ± 10.2; 30 min, 10.6 ± 5.2 and 16.5 ± 8.3, P < 0.001 for both). There was a slight increase in systolic pressure and heart rate after three and four minutes of the infusion with normalization of these parameters thereafter. Patients showed good tolerance to MnCl_2 _with no major adverse events, despite all reporting transient facial flush.

**Conclusions:**

In the short term, MnCl_2 _appears safe for human use. It effectively decreases myocardium T_1_, maintaining this effect for a relatively long period of time and allowing for the development of new imaging strategies in CMR, especially in ischemia research.

## Background

Cardiovascular magnetic resonance (CMR) has become one of the most important diagnostic tools in cardiology, especially in infarct detection and myocardial perfusion in coronary artery disease [1,2]. Gadolinium contrast enhanced CMR is frequently used for that purpose with a relatively good safety profile but has been recently associated with systemic nephrogenic fibrosis [3]. Moreover, due to their extracellular distribution, gadolinium chelates are rapidly washed out of myocardial tissue, limiting the spatial and temporal resolution as well as the volume coverage that can be obtained in CMR studies [4]. Intracellular paramagnetic agents that are retained inside myocytes for longer periods may circumvent these limitations and offer an increase in diagnostic accuracy.

In solution, MnCl2 freely dissociates into Mn2^+ ^and Cl^- ^ions. The manganese ions, which are strongly paramagnetic, behave in vivo as a calcium analogue, and actively enter myocytes through voltage-gated calcium channels [5,6]. Once inside myocytes, manganese is retained within intracellular organelles such as mitochondria, remaining in the cell for several days. In animals, experimental studies showed that the use of MnCl_2 _allows for the detection of ischemic tissues as well as infarcted areas with great accuracy [7-9]. However, due to competition with calcium for voltage-gated channels there has always been a safety concern regarding the use of manganese in high concentrations because of the possibility of acute heart failure or even cardio-pulmonary arrest [10]. Despite animal studies having shown that this concern does exist in extremely high doses, at lower concentrations MnCl_2 _has not been associated with any serious adverse short term events [11]. The use other manganese compounds like manganese dipyridoxyl-diphosphate (MnDPDP) has been done in humans before with a good safety profile [12]. However, because MnCl_2 _releases Mn2^+ ^much more rapidly than MnDPDP allowing for more rapid uptake of the free manganese ions by myocytes, it may in one hand promote deleterious effects on heart function due to direct competition with Ca2^+ ^with possible decrease in inotropism [13] at the same time providing for potential advantages in cardiac imaging techniques due to its long lasting intracellular properties.

Based on these observations, the purpose of this study was to investigate the safety and efficacy of a rapid infusion of MnCl_2 _for CMR in humans. We found that low-dose MnCl_2 _appears to offer a safe profile with significant enhancement of myocardial tissue in different pulse sequence protocols.

## Methods

### Study Population

Fifteen healthy volunteers of both sexes with ages from 18 to 60 were prospectively selected for the study. Inclusion criteria consisted of absence of previous cardiovascular disease or risk factors (hypertension, dyslipidemia, diabetes, acute myocardial infarction, angina pectoris, valvular disease) and normal left ventricular function (normal chest radiograph or echocardiography with an ejection fraction > 0.60 without symptoms of heart failure). We excluded from the study individuals taking any chronic medication, current smokers, and those with a history of liver diseases, arrhythmias detected in a resting electrocardiogram (ECG) or with known absolute contra-indications for undergoing a CMR study.

### Study Protocol

After the initial ECG to assess PR, QRS and QT intervals, subjects underwent a CMR exam without any contrast in a 1.5 T scanner (Siemens Symphony, Erlangen, Germany) with a four-element phased-array coil. After localizer sequences, cine images for ventricular function evaluation were obtained using previously described steady-state free-precession (SSFP) sequences in short axis, two-chamber and four-chamber views. After that, a short axis mid-ventricular slice was chosen and four different imaging protocols acquired: (1) 2D gradient-echo inversion recovery (GRE-IR) sequence (TR 6.0 ms; TE 4.18 ms; matrix 256 × 128; FOV 350 × 260; resolution 1.37 × 2.0 × 8 mm; FA 25°; BW 130 Hz/px; TI 400 ms; 25 segments; 2 RR intervals); (2) 3D GRE-IR sequence (TR 3.8 ms; TE 1.55 ms; matrix 256 × 102; FOV 350 × 260; resolution 1.37 × 2.55 × 5 mm; FA 10°; BW 360 Hz/px; TI 400 ms; 51 segments; 1 RR interval); (3) phase-sensitive IR sequence (same parameters as the 2D GRE-IR) and (4) a single breath-hold segmented IR prepared steady-state precession (sIR-SSFP) acquisition for T_1 _calculations (TR 26.7 ms; TE 1.27 ms; matrix 192 × 72; FOV 350 × 260; resolution 1.82 × 3.61 × 8 mm; FA 50°; BW 965 Hz/px; TI variable; 9 segments; 2 RR intervals).

After acquiring these images, subjects were removed from inside the magnet without moving from the table. Venous access was obtained and 5 μMol/kg of MnCl_2 _was manually infused over three minutes (1.67 μMol/kg/min). MnCl_2 _was manufactured in 10 ml vials of 20 mg/mL (Farmacia do Hospital das Clinicas da USP, Sao Paulo, Brazil) and the total amount of MnCl_2 _per subject was calculated, drawn from the vial and diluted to 10 mL with normal saline. During and ten minutes after the infusion, heart rate, arterial pressure and any arrhythmias were recorded (Maglife C Plus, Schiller Medical, Switzerland). Subjects were questioned regarding symptoms throughout the study.

Immediately after the infusion, subjects entered the scanner once again and the same single slice images were obtained using the four previously described sequences with cine images being acquired shortly after that. At fifteen and thirty minutes after the end of the infusion, new single slice images were again obtained as described, with new cine images at thirty minutes only. At the end of the study, a final ECG was performed before the subject left the facility. After six months, all subjects were contacted by telephone and questioned about the occurrence of clinical events that included: death by any cause, myocardial infarction, angina symptoms (mainly typical chest pain), heart failure symptoms (dyspnea, lower limb edema, fatigue) and neurological disturbances (gait changes, tremors, motor and sensitive deficits).

### Ethical Issues

The local institutional review board approved the study methods and all subjects provided written informed consent. Since this was the first reported use of MnCl_2 _in humans, great care was taken to assure safety and ethical considerations in applying the drug. The local ethics committee required that the lowest dose of the drug was to be used (5 μMol/kg) since this concentration of manganese had already been used in humans with the commercially approved contrast MnDPDP. This dose along was chosen along with an infusion rate significantly lower than the reported doses shown to cause any adverse effects on heart function in animals. The ethics committee therefore understood that this new agent would provide a total dose of manganese similar to the one approved for MnDPDP while releasing Mn2^+ ^more rapidly but well below a perilous rate as previously described. Finally, patients were closely monitored before, during and after the infusions and stayed in the lab for two hours after the procedure. We also took the care of contacting each individual six months after the infusion to assess any potential complications not predicted in the day of the CMR study.

### Image Analysis

All images were anonymized prior to analysis for subject name and timing of the study. All images were analyzed by the same reader (JLF, six years of experience in CMR), in random order. The choice of a single reader for all images was done due to the blind reading form described for image analysis as well as for the rather objective values obtained from this analysis, requiring very little subjective assessment. Cine images were evaluated for diastolic volume, systolic volume and ejection fraction using commercial software (Argus Syngo MR2004A, Siemens, Germany). Volumes were determined semi-automatically by drawing endocardial borders in each slice at systole and diastole from base to apex excluding papillary muscles according to established references [14]. For signal intensity changes before and at different time points after infusion of MnCl_2_, a manual region of interest (ROI) was drawn including all the myocardium for each of the pulse sequences and the mean signal intensity was chosen for each ROI. Signal to noise ratio (SNR) was calculated as the ratio between the ROI mean and the standard deviation of background noise. Contrast to noise ratio (CNR) was calculated as the difference between precontrast SNRs and each postcontrast values [15]. To calculate T1 and R1 (1/T1), we used the data from the sIR-SSFP sequence with multiple inversion times, fitting the curve using customized programs written in Matlab (Mathworks Inc, USA) according to previously published methods [16].

### Statistical Analysis

Sample size calculation was not performed previous to the study; rather, the number of subjects was selected according to other similar studies with MnCl_2 _in animals which used from eight to thirty subjects [7,17,18]. We compared CNR and SNR data, T1 and R1 values, heart rate, arterial pressure, ventricular function data and ECG parameters using analysis of variance for repeated measures with Statview 5.0 (SAS Institute, USA) with LSD post-hoc test as needed. A value of P < 0.05 was considered statistically significant.

## Results

All the 15 subjects selected (age 46 ± 11 years, five men) completed the study. No subject suffered any major adverse effect during the CMR exam or within six months of completing the study.

### Efficacy

After MnCl_2 _infusion there was a significant increase in mean SNR in all sequences analyzed except in the sIR-SSFP sequence (Table 1). Myocardium T1 values also decreased significantly immediately after the infusion, maintaining this effect almost uniformly throughout thirty minutes.

**Table 1:** Signal to noise ratio and T1/R1 values at baseline, immediately, 15 and 30 minutes after the infusion of MnCl_2 _using different sequences.

Variable	Pre	0 min	15 min	30 min	P
GRE-IR 2D	6.6 ± 4.2	11.3 ± 4.1	10.8 ± 4.0	10.6 ± 5.2	< 0.0001
GRE-IR 3D	9.7 ± 5.3	15.0 ± 8.7	16.9 ± 10.2	16.5 ± 8.3	< 0.001
IR-SSFP	10.7 ± 4.2	12.8 ± 4.2	11.5 ± 2.4	12.7 ± 2.5	0.16
Phase Sensitive IR	13.5 ± 3.0	16.3 ± 3.9	15.9 ± 4.2	14.6 ± 3.6	0.02
T_1 _(ms)	839 ± 281	684 ± 99	714 ± 168	706 ± 172	0.003
R1 (Hz)	1.32 ± 0.46	1.50 ± 0.24	1.47 ± 0.34	1.50 ± 0.39	0.02

GRE-IR = gradient-echo inversion recovery; SSFP = steady-state free precession

Data represents mean ± SD

**Figure 1:**
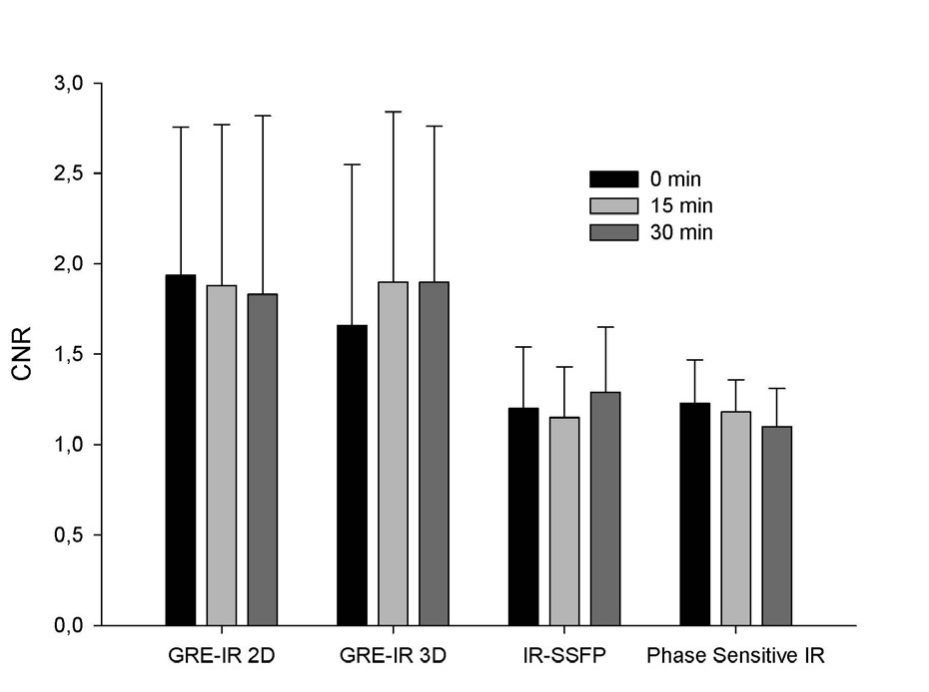
**Contrast to noise (CNR) values at different time points after the infusion of MnCl**_**2 **_**using different sequences**. Both 2D and 3D gradient-echo inversion recovery (GRE-IR) sequences showed the greatest increase in CNR compared to baseline than Phase contrast inversion recovery (IR) and IR steady state free precession (SSFP). There were no significant CNR differences among each sequence immediately, at 15 or 30 minutes after the infusion of MnCl_2_.

**Figure 2:**
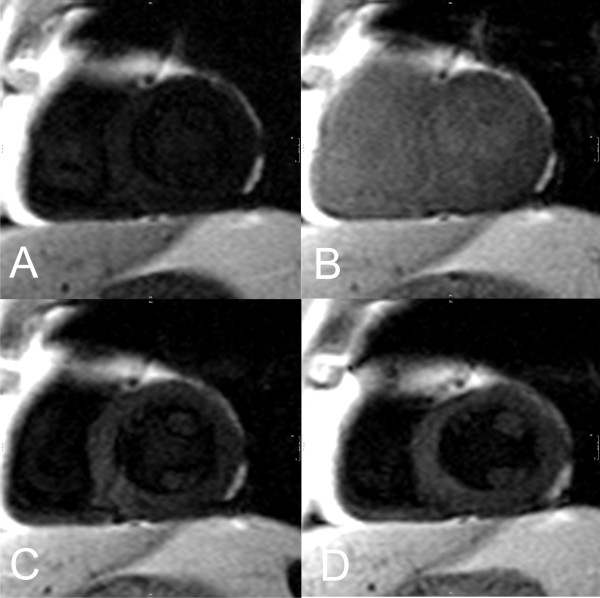
**Signal changes in a typical subject using the 2D gradient-echo inversion recovery 2D sequence**: (A) pre contrast image; (B) immediately after the infusion; (C) 15 minutes after; (D) 30 minutes after.

**Figure 3:**
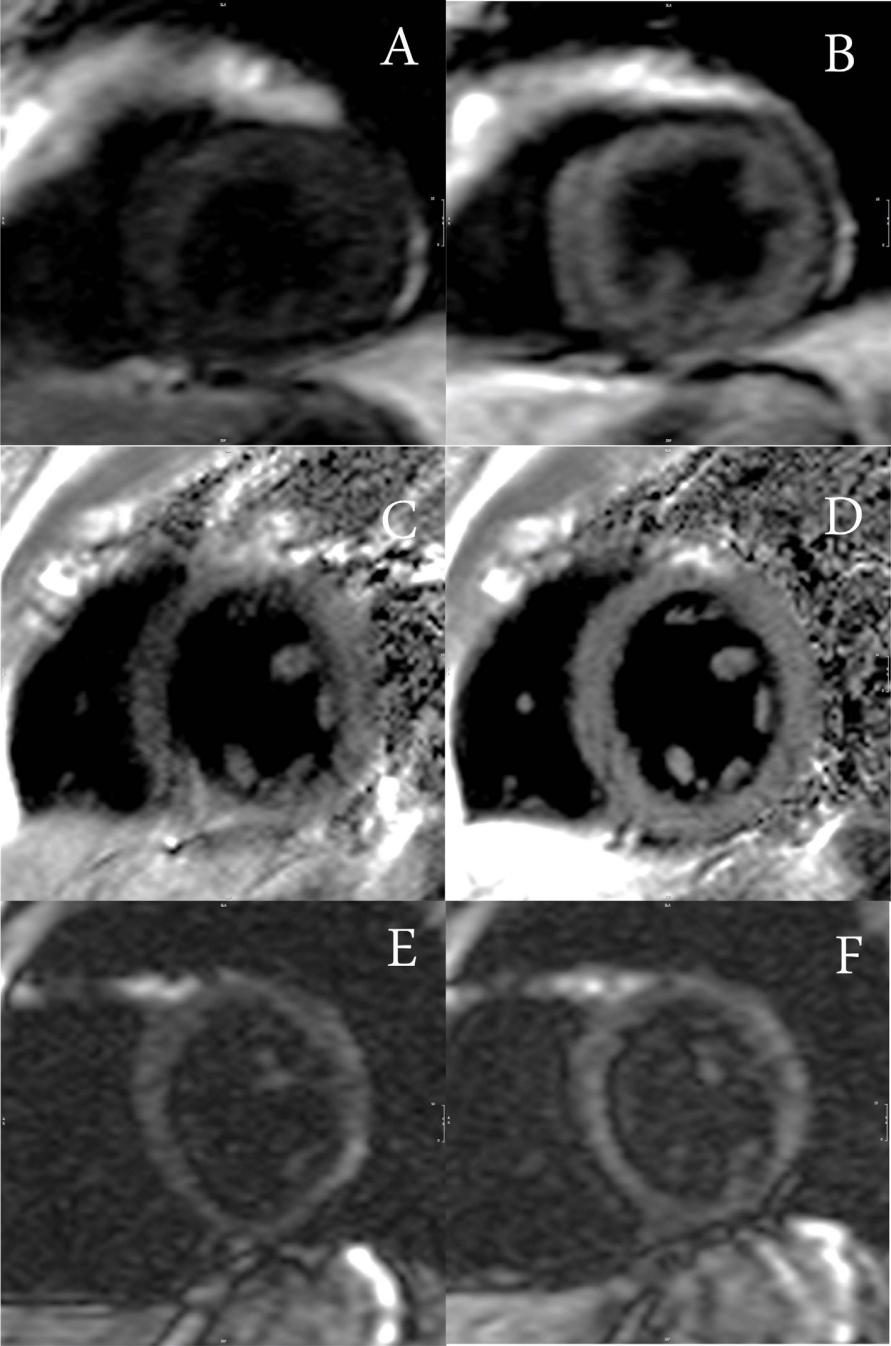
**Signal changes pre and 30 minutes after the infusion of MnCl_2 _using different sequences in three different patients**: (A) and (B) gradient-echo inversion recovery 3D; (C) and (D) Phase contrast inversion recovery; (E) and (F) steady state free precession.

To evaluate if the changes observed stayed up to 30 minutes after the infusion in the different sequences used and if signal intensity increase differed among them, we compared CNR values for each sequence in different time points (Figure 1). No significant differences were found among each time point supporting the findings of the T1 values that the increase in signal persists for up to 30 minutes after infusion (P = 0.52). Regarding the comparison among the different sequences, we found that GRE-IR 2D and GRE-IR 3D demonstrated significant increases in CNR compared to IR-SSFP (P = 0.015 and P = 0.03 respectively) and phase sensitive IR (P = 0.008 and P = 0.017 respectively) almost doubling the baseline SNR values. While phase sensitive IR also showed a significant increase in SNR compared to baseline this increase was not as prominent as the observed with the GRE-IR sequences. An example of images at all time points with the GRE-IR 2D sequence is shown in Figure 2. An example of images pre and 30 minutes after infusion of MnCl_2 _with the other sequences are shown in Figure 3.

### Short Term Safety

**Figure 4:**
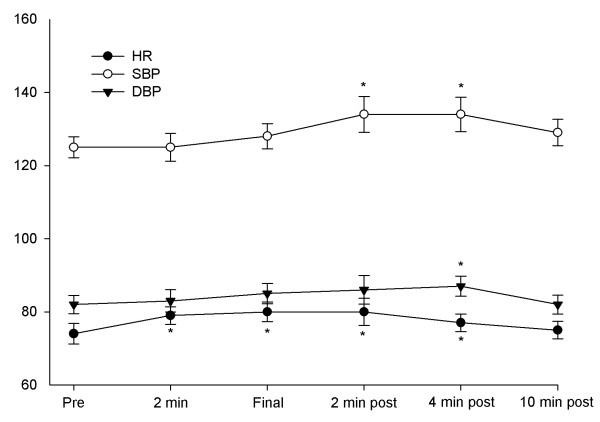
**Arterial blood pressure and heart rate variations during and after the infusion of MnCl_2_**. * P < 0.05 compared to pre infusion values.

A small but significant transient increase in arterial systolic and diastolic pressure was observed after the infusion of MnCl_2_, with maintenance of this effect up to four minutes after the drug was injected (Figure 4). At ten minutes, both pressures had returned to baseline values. While significant, blood pressure values kept within the normal range during the whole study. The heart rate also showed a small but significant elevation from 74.1 ± 10 beats per minute at baseline to a maximum of 80.2 ± 9.8 at the end of the infusion (P = 0.002). Changes in heart rate were already observed after two minutes of the infusion and disappeared at ten minutes after drug injection was completed. Pre infusion ECG showed a PR and QRS duration of 139.2 ± 15.0 and 80.5 ± 19.3 msec respectively without any significant differences compared to post infusion ECG of 141.2 ± 16.5 and 78.9 ± 22.2 (P = 0.99 and 0.55 respectively). Corrected QT duration was also not changed comparing pre and post measures (398.8 ± 26.7 versus 405.5 ± 29.3, P = 0.39).

One of the main concerns regarding the use of MnCl_2 _in humans was a significant change in ventricular function due to competition of manganese with calcium for entering myocytes. This was not seen with this dose and speed of infusion of MnCl_2 _(table 2). No significant differences were observed after the infusion of the drug or after thirty minutes regarding left ventricular ejection fraction, diastolic and systolic volumes.

**Table 2:** Changes in ejection fraction and cardiac volumes compared to baseline immediately, 15 and 30 minutes after MnCl_2 _infusion.

Variable	Pre	0 min	30 min	P
Ejection Fraction (%)	65.7 ± 1.7	67.1 ± 1.6	67.0 ± 1.9	0.22
Systolic volume (mL)	39.8 ± 4.3	38.3 ± 3.6	37.1 ± 4.1	0.24
Diastolic Volume (mL)	114.0 ± 8.4	120 ± 11.2	110.0 ± 8.3	0.12

Finally, subjects did not report experiencing any serious adverse symptoms during or after the infusion of MnCl_2, _demonstrating good tolerance for the drug. However, after two minutes of infusion, all subjects reported feeling a transient facial flush that lasted up to two minutes after stopping the infusion. This effect did not prevent any subject from completing the study. On the six month follow-up, no patient reported any pre-specified neurologic or cardiologic adverse event or symptom.

## Discussion

Manganese has been described in the past as a promising paramagnetic contrast agent and was tested in parallel to gadolinium [13]. When administered in an unchelated form, such as MnCl_2_, manganese has a short plasma half-life with 99% of free Mn2^+ ^being cleared from plasma within 2.7 minutes [19]. Its distribution is almost entirely intracellular, where it is retained mainly within mitochondria with very slow recirculation [20]. This property was previously observed in animal models where changes T1 lasted up to 2 hours after infusion of the drug [7]. In our study, we tested this effect in humans and similar results were found with a significant decrease in longitudinal relaxation, which lasted at least thirty minutes after the infusion. In our view, this first human experiment with the drug opens many opportunities to further explore myocardium perfusion uptake and calcium activity under various physiological and pathological conditions, although further optimization of sequence protocols will be needed to differentiate tissue with varying degrees of manganese uptake. Differences between normal and ischemic myocardial tissue have been identified in dogs previously [18] and this approach might be reproducible in humans if the decrease in T1 values observed are large enough with the dose used in the study, allowing for possible infusion of MnCl_2 _outside the magnet and acquisition of images at rest under steady state conditions using higher resolution sequences, similar to a nuclear thallium test [21].

However, to be useful for human studies, it is mandatory that safety issues are taken into account since previous animal studies have shown that in very large doses manganese can induce acute heart failure [22]. In our study, we chose the total dose of 5 μMol/kg of MnCl_2 _with an infusion rate of 1.67 μMol/kg/min. Animal studies have used variable doses from 1 to 420 μMol/kg (with most studies limiting the dose from 5 to 30 μMol/kg) [23] with infusion rates of most commonly within the range of 1 to 15 μMol/kg/min [17,18,24,25]. Only studies with very high infusion rates (generally over 100 μMol/kg/min) have shown serious adverse effects in heart rate or arterial pressure [11]. In our study, blood pressure did not change significantly during the infusion of manganese with a slight increase in heart rate possibly compensating any negative inotropic provoked by the ion. The apparent discrepancy with previous data might be related to the differences in doses used since in this study a lower dose of MnCl2 was infused. In that regard, the infusion rate of MnCl_2 _seems to be much more important regarding acute safety than the total dose used since free manganese directly competes with calcium for voltage channels in the heart. At the same time, if one wants to look at uptake by myocytes during ischemia (for example, at the peak of a treadmill test), it is important to guarantee a very fast delivery of Mn2^+ ^to the heart. Having that in mind, our data suggests that the infusion rate observed in our study guarantees a short term safety profile without significant clinical changes in heart rate, arterial pressure and ventricular function in patients without heart disease, while still permitting delivery of MnCl_2 _at a rate where possible ischemia detection might prove achievable. Whether higher infusion rates or total doses of the drug might exhibit the same safety profile with better efficacy remains to be shown.

The only other manganese based contrast agent to have been used in humans previously for the study of the heart is MnDPDP, which is a chelated agent, and has different pharmacokinetics and biological distribution from MnCl_2 _[26-30]. With MnDPDP using similar concentrations of manganese as our study, the authors demonstrated a R1 increase of 0.38 ± 0.03 seconds in non-infarcted areas at one hour, higher than our finding of 0.26 ± 0.06 seconds at thirty minutes. This might have been achieved by different properties of MnDPDP to release free manganese or recirculation of Mn2^+^. Despite that, the delivery of Mn2^+ ^to myocytes using MnDPDP is very slow while MnCl_2 _promotes rapid availability of the ion, a potential advantage especially for stress ischemia studies. Nevertheless, if MnCl_2 _is to be used in humans over the established MnDPDP remains to be proven in a larger study in patients with ischemic heart disease. The other manganese compound used in humans was EVP 1001-1, a compound with a short vascular half-life of 1.5 minutes with flow-dependent differences in uptake similar to the uses proposed for MnCl_2 _[26,27]. This compound is being tested in a Phase II human trial without any results reported yet (NCT00340925) and might provide some of the advantages observed with MnCl_2 _with an adequate safety profile as well.

### Limitations on safety assessment

This study has many safety limitations and these have to be taken into account when interpreting our data. First, the main objective of this study was to assess the acute effects of free Mn2^+ ^to the heart. Therefore, we did not evaluate other possible effects of MnCl_2 _on the liver or kidneys, did not measure the concentration of the ion in the blood or urine or its interaction with iron or copper metabolism. While we did make contact with the subjects six months after the exam regarding clinical consequences of manganese toxicity on other organs, the number of subjects studied and the period of observation do not allow us to reach any conclusions regarding long-term safety. A publication studying methcathinone users with high levels of whole blood manganese has shown that the mean time between extrapyramidal symptoms (a known effect of chronic manganese intoxication [31]) and drug use was 5.8 years [32]. There are rare descriptions of acute intoxication by manganese despite its frequent use in industry with most of the cases being described after long years of chronic exposure to the substance [33,34]. Nevertheless, this issue was not addressed in our study.

Another limitation of this study is that we only looked at normal individuals without left ventricular dysfunction or ischemic heart disease. It is not known if the safety and efficacy data presented here can be reproduced in patients who already have borderline cardiac function or previous ischemia. While the main cardiotoxicity of manganese is related more to the extracellular than the intracellular concentration of Mn2^+ ^[35] the possibility of evaluating cardiac function at a later time would have been wise.

Finally, this preliminary data might be considered limited to further advance the use of substance in humans, especially in some countries where other safety measures would be required to allow for subsequent trials with MnCl_2_.

The study was limited to a fixed infusion rate and total amount of MnCl_2_, preventing us from addressing whether higher doses would be tolerated, more efficient or even allowing for the study of concentration and signal enhancement relationships. Nevertheless, it had been shown with MnDPDP that increasing the dose to 10 or 15 μMol/kg did not yield further myocardial enhancement thus only allowing for an increase in side effects [30]. Finally, while we could also have obtained more delayed (> 30 min) imaging points, we thought that this would deviate from the potential clinical applications suggested by the agent as well as not be practical in routine human scans or useful for assessment of safety issues.

## Conclusions

In conclusion, our data suggests that low-dose MnCl_2 _(5 μMol/kg) has a good short-term cardiac safety profile in humans promoting a significant decrease in myocardium T1 for a relatively long time. From this data, we believe that new strategies to study coronary heart disease with CMR can be designed with special focus on ischemia with higher resolution sequences and out of the magnet protocols, taking advantage of the longer-lasting changes in relaxation times produced by the agent.

## Competing interests

The authors declare that they have no competing interests.

## Authors' contributions

JLF has participated in all phases of the study including its design, acquisition and interpretation of data and drafting of the manuscript; PS participated in the interpretation of data and drafting of the manuscript; JAS, GSF and JMK participated in the acquisition of data; ORC has participated in the study design, interpretation of data and drafting of the manuscript. All authors have read approved the final manuscript.

## References

[B1] Lima JA Desai MY Cardiovascular magnetic resonance imaging: current and emerging applications J Am Coll Cardiol 2004 44 1164 1171 10.1016/j.jacc.2004.06.033 15364314

[B2] Shan K Constantine G Sivananthan M Flamm SD Role of cardiac magnetic resonance imaging in the assessment of myocardial viability Circulation 2004 109 1328 1334 10.1161/01.CIR.0000120294.67948.E3 15037539

[B3] Thomsen HS Marckmann P Logager VB Update on nephrogenic systemic fibrosis Magn Reson Imaging Clin N Am 2008 16 551 560 vii 10.1016/j.mric.2008.07.011 18926421

[B4] Schneider G Fries P Ahlhelm F Kindermann I Kramann B Bohm M Contrast-enhanced cardiac MR imaging Eur Radiol 2003 13 Suppl 3 N11 18 10.1007/s00330-003-0002-4 15015876

[B5] Lee JH Koretsky AP Manganese enhanced magnetic resonance imaging Curr Pharm Biotechnol 2004 5 529 537 10.2174/1389201043376607 15579042

[B6] Koretsky AP Silva AC Manganese-enhanced magnetic resonance imaging (MEMRI) NMR Biomed 2004 17 527 531 10.1002/nbm.940 15617051

[B7] Natanzon A Aletras AH Hsu LY Arai AE Determining canine myocardial area at risk with manganese-enhanced MR imaging Radiology 2005 236 859 866 10.1148/radiol.2363040413 16118166

[B8] Saeed M Wendland MF Watzinger N Akbari H Higgins CB MR contrast media for myocardial viability, microvascular integrity and perfusion Eur J Radiol 2000 34 179 195 10.1016/S0720-048X(00)00198-4 10927160

[B9] Bremerich J Saeed M Arheden H Higgins CB Wendland MF Normal and infarcted myocardium: differentiation with cellular uptake of manganese at MR imaging in a rat model Radiology 2000 216 524 530 10924581 10.1148/radiology.216.2.r00jl14524

[B10] Federle MP Chezmar JL Rubin DL Weinreb JC Freeny PC Semelka RC Brown JJ Borello JA Lee JK Mattrey R Safety and efficacy of mangafodipir trisodium (MnDPDP) injection for hepatic MRI in adults: results of the U.S. multicenter phase III clinical trials (safety) J Magn Reson Imaging 2000 12 186 197 10.1002/1522-2586(200007)12:1<186::AID-JMRI21>3.0.CO;2-2 10931579

[B11] Wolf GL Baum L Cardiovascular toxicity and tissue proton T1 response to manganese injection in the dog and rabbit AJR Am J Roentgenol 1983 141 193 197 6305179 10.2214/ajr.141.1.193

[B12] Federle M Chezmar J Rubin DL Weinreb J Freeny P Schmiedl UP Brown JJ Borrello JA Lee JK Semelka RC Efficacy and safety of mangafodipir trisodium (MnDPDP) injection for hepatic MRI in adults: results of the U.S. Multicenter phase III clinical trials. Efficacy of early imaging J Magn Reson Imaging 2000 12 689 701 10.1002/1522-2586(200011)12:5<689::AID-JMRI5>3.0.CO;2-Z 11050638

[B13] Slutsky RA Peterson T Strich G Brown JJ Hemodynamic effects of rapid and slow infusions of manganese chloride and gadolinium-DTPA in dogs Radiology 1985 154 733 735 3969479 10.1148/radiology.154.3.3969479

[B14] Maceira AM Prasad SK Khan M Pennell DJ Normalized left ventricular systolic and diastolic function by steady state free precession cardiovascular magnetic resonance J Cardiovasc Magn Reson 2006 8 417 426 10.1080/10976640600572889 16755827

[B15] Firbank MJ Coulthard A Harrison RM Williams ED A comparison of two methods for measuring the signal to noise ratio on MR images Phys Med Biol 1999 44 N261 264 10.1088/0031-9155/44/12/403 10616158

[B16] Schmitt P Griswold MA Jakob PM Kotas M Gulani V Flentje M Haase A Inversion recovery TrueFISP: quantification of T(1), T(2), and spin density Magn Reson Med 2004 51 661 667 10.1002/mrm.20058 15065237

[B17] Flacke S Allen JS Chia JM Wible JH Periasamy MP Adams MD Adzamli IK Lorenz CH Characterization of viable and nonviable myocardium at MR imaging: comparison of gadolinium-based extracellular and blood pool contrast materials versus manganese-based contrast materials in a rat myocardial infarction model Radiology 2003 226 731 738 10.1148/radiol.2263020151 12601183

[B18] Hu TC Christian TF Aletras AH Taylor JL Koretsky AP Arai AE Manganese enhanced magnetic resonance imaging of normal and ischemic canine heart Magn Reson Med 2005 54 196 200 10.1002/mrm.20516 15968667

[B19] Mahoney JP Small WJ Studies on manganese. 3. The biological half-life of radiomanganese in man and factors which affect this half-life J Clin Invest 1968 47 643 653 5637148 10.1172/JCI105760 PMC297210

[B20] Maynard LS Cotzias GC The partition of manganese among organs and intracellular organelles of the rat J Biol Chem 1955 214 489 495 14367406

[B21] Wendland MF Saeed M Bremerich J Arheden H Higgins CB Thallium-like test for myocardial viability with MnDPDP-enhanced MRI Acad Radiol 2002 9 Suppl 1 S82 83 10.1016/S1076-6332(03)80405-1 12019904

[B22] Brady TJ Goldman MR Pykett IL Buonanno FS Kistler JP Newhouse JH Burt CT Hinshaw WS Pohost GM Proton nuclear magnetic resonance imaging of regionally ischemic canine hearts: effect of paramagnetic proton signal enhancement Radiology 1982 144 343 347 6283594 10.1148/radiology.144.2.6283594

[B23] Wendland MF Krombach GA Higgins CB Novikov V Saeed M Contrast enhanced MRI of stunned myocardium using Mn-based MRI contrast media Acad Radiol 2002 9 Suppl 2 S341 342 10.1016/S1076-6332(03)80224-6 12188269

[B24] Hu TC Pautler RG MacGowan GA Koretsky AP Manganese-enhanced MRI of mouse heart during changes in inotropy Magn Reson Med 2001 46 884 890 10.1002/mrm.1273 11675639

[B25] Eriksson R Johansson L Bjerner T Ahlstrom H Dobutamine-induced stress affects intracellular uptake of manganese: a quantitative magnetic resonance imaging study in pigs J Magn Reson Imaging 2005 21 360 364 10.1002/jmri.20279 15779028

[B26] Storey P Chen Q Li W Seoane PR Harnish PP Fogelson L Harris KR Prasad PV Magnetic resonance imaging of myocardial infarction using a manganese-based contrast agent (EVP 1001-1): preliminary results in a dog model J Magn Reson Imaging 2006 23 228 234 10.1002/jmri.20500 16416440

[B27] Storey P Danias PG Post M Li W Seoane PR Harnish PP Edelman RR Prasad PV Preliminary evaluation of EVP 1001-1: a new cardiac-specific magnetic resonance contrast agent with kinetics suitable for steady-state imaging of the ischemic heart Invest Radiol 2003 38 642 652 10.1097/01.rli.0000077057.88108.3f 14501492

[B28] Skjold A Amundsen BH Wiseth R Stoylen A Haraldseth O Larsson HB Jynge P Manganese dipyridoxyl-diphosphate (MnDPDP) as a viability marker in patients with myocardial infarction J Magn Reson Imaging 2007 26 720 727 10.1002/jmri.21065 17729351

[B29] Skjold A Kristoffersen A Vangberg TR Haraldseth O Jynge P Larsson HB An apparent unidirectional influx constant for manganese as a measure of myocardial calcium channel activity J Magn Reson Imaging 2006 24 1047 1055 10.1002/jmri.20736 17024667

[B30] Skjold A Vangberg TR Kristoffersen A Haraldseth O Jynge P Larsson HB Relaxation enhancing properties of MnDPDP in human myocardium J Magn Reson Imaging 2004 20 948 952 10.1002/jmri.20200 15558550

[B31] Mergler D Neurotoxic effects of low level exposure to manganese in human populations Environ Res 1999 80 99 102 10.1006/enrs.1998.3902 10092399

[B32] Stepens A Logina I Liguts V Aldins P Eksteina I Platkajis A Martinsone I Terauds E Rozentale B Donaghy M A Parkinsonian syndrome in methcathinone users and the role of manganese N Engl J Med 2008 358 1009 1017 10.1056/NEJMoa072488 18322282

[B33] Dickerson RN Manganese intoxication and parenteral nutrition Nutrition 2001 17 689 693 10.1016/S0899-9007(01)00546-9 11448607

[B34] Olanow CW Manganese-induced parkinsonism and Parkinson's disease Ann N Y Acad Sci 2004 1012 209 223 10.1196/annals.1306.018 15105268

[B35] Wendland MF Applications of manganese-enhanced magnetic resonance imaging (MEMRI) to imaging of the heart NMR Biomed 2004 17 581 594 10.1002/nbm.943 15761947

